# Nutritional, pharmaceutical, and functional aspects of rambutan in industrial perspective: An updated review

**DOI:** 10.1002/fsn3.3379

**Published:** 2023-04-19

**Authors:** Muhammad Afzaal, Farhan Saeed, Maryam Bibi, Afaf Ejaz, Yasir Abbas Shah, Zargham Faisal, Huda Ateeq, Noor Akram, Aasma Asghar, Mohd Asif Shah

**Affiliations:** ^1^ Food Safety and Biotechnology lab, Department of Food Science Government College University Faisalabad Faisalabad Pakistan; ^2^ Institute of Food Science and Nutrition Bahauddin Zakariya University Multan Multan Pakistan; ^3^ Department of Home Economics Government College University Faisalabad Faisalabad Pakistan; ^4^ Department of Economics, College of Business and Economics Kebri Dehar University Kebri Dehar Somali Ethiopia; ^5^ Division of Research and Development Lovely Professional University Phagwara Punjab India

**Keywords:** antioxidant, functional food, industrial applications, phytochemical, rambutan

## Abstract

Numerous researchers have been motivated to investigate new plant sources as a result of the continued advancement of functional foods and herbal medicines. The rambutan fruit (*Nephelium lappaceum* L.) with its significant nutritional and bioactive compositions offers therapeutic properties such as anticancer, antiallergic, antiobesity, antidiabetic, antimicrobial, antihypercholesterolemic, and antihyperglycemic. Rambutan is high in antioxidants, dietary fibers, and vitamins and minerals. Its parts including fruit peel, pulp, and seed are a great source of bioactive compounds. Rambutan fruit extracts have been found to have cardioprotective and hepatoprotective properties. This review provides an insight into the nutritional as well as therapeutic value, health potential, and utilization of rambutan fruit along with its nonedible parts (seeds and peels). Advanced research and phytochemical screening would also encourage the rambutan fruit as a viable choice for the preparation of medications and functional foods. However, it is necessary to further analyze the functional aspects and nutritional potential of this fruit along with the therapeutic mechanisms and to improve its industrial use as a nutraceutical and functional food product.

## Graphical abstract

Rambutan contains a number of bioactive components in its peel, seed, and pulp. Phytochemical substances have nutritional properties and therapeutic potential such as antioxidant, antibacterial, anticancer, antidiabetic, antiviral, anti‐inflammatory, and antiproliferative activities. To improve this fruit's industrial processing as a functional or medicinal food product, the underlying medicinal strategies as well as its nutritional and physiological potential to be further assessed.

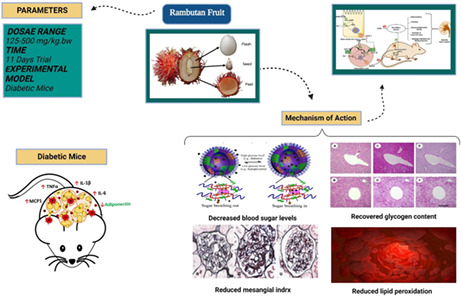

## 1 INTRODUCTION

Rambutan (*Nephelium lappaceum* L.) fruits are tropical fruits, edible, oval to spherical in shape, have leathery skin texture with flexible hairy spines, and are commonly red in color (Minh et al., 2019). Rambutan grows naturally in several tropical parts of Southeast Asia including Indonesia and Malaysia; even though, commercial improvement has expanded to Singapore, the Philippines, Syria, Thailand, Australia, India, Madagascar, and Congo (Mahmood et al., 2018). It is linked to the family of subtropical fruits, Sapindaceae, pulasan (*Nephelium mutabile* Blume) lychee (*Litchi chinensis* Sonn), and longan (*Euphoria longan* Steud). Indonesia, Malaysia, and Thailand are the biggest cultivators of rambutan fruit and they export it to other countries (Jahurul et al., 2020).

A medium‐sized evergreen tree (11–21 m) of rambutan has bright to dark green compound leaves with (2–10) leaflets, and each varies in length and breadth from 4 to 16 cm. Rambutan has the following composition 26.4% of total weight, 14.2% peel, 2.59% seed, 12.1% pulp, and 1.56% embryo on dry basis. The fruit contains pulp having a juicy, glossy, and whitish appearance having a slight tangy flavor. The pulp‐covered seed is ovoid or rectangular, with dimensions of 2.5–3.41–1.5 cm. Rambutan is a nonclimacteric fruit and therefore it can be plucked as it ripens (Hernández‐Hernández et al., 2019). Color change variation considers the crucial aspect of cultivated ripeness in the case of red rambutan fruit. For example, a lower limit of 16% of dissolved solids could indicate the maturity stage of the fruit. Additionally, it should differ significantly until maturity, varying between cultivars by 16%–22% (Palanisamy et al., 2008). The fruit weight is between 22.4 and 64.7 g depending on cultivar and agronomical circumstances. The rambutan fruit is typically eaten fresh because of its vibrant color, beautiful look, and tart flavor. This fruit can be used to cure fever, diarrhea, and digestive problems. Rambutan skin in dried form is used as conventional medicine in Malaysia and other countries. Rambutan leaves can be used as a bandage to ease headaches, while the skin could be used as an astringent. Its roots are commonly consumed to reduce fever (Palanisamy et al., 2008).

According to recent reports, Thailand cultivated 319,000 tons of rambutan fruit from 2014 to 2015. The fruit is rich in carbohydrates, proteins, vitamins, minerals, and antioxidants with greater flavor. Rambutan juice, chips, marmalades, jams, jelly spreads, and bottled in syrups are some of the most popular and fresh rambutan items in Malaysia (Jahurul et al., 2020). The rambutan primarily consists of seeds and peels that are thrown as by‐products of direct eating or industrial processing. Recently, an estimated 1900 tons of rambutan seeds are thrown as a by‐product in Thailand each year. A large amount of this industrial by‐product, if not used efficiently, creates major environmental problems and economic losses (Chai et al., 2018). The advantages of rambutan fruit and their by‐products in terms of their nutritional and therapeutic potential in the fields of health are being analyzed. However, rambutan fruit rapidly loses its attractive appearance after harvesting period because of its superficial pericarp browning which appeared to affect rambutan pericarp tissue in much the same manner as senescence (Shahrajabian et al., 2020).

## 2 NUTRITIONAL COMPOSITION OF RAMBUTAN FRUIT

Chigurupati et al. (2019) worked on the nutritional composition of rambutan and reported the presence of total flavonoid contents and total phenolic content in terms of gallic acid and rutin equivalents; however, antioxidant function of rambutan inhibited α‐amylase, α‐glucosidase enzyme activities and inhibition of ABTS radicals along with DPPH activity. The biochemical composition of fruit is indicated in Table 1.

**TABLE 1:** Nutritional composition of rambutan fruit.

Components	Rambutan fruit peel	Rambutan fruit pulp	Rambutan fruit raw seed	References
Carbohydrates (g 100 g^−1^)	22.73	33.63–61.5	15.5–18.99	Hernández‐Hernández et al. (2019)
Lipids (g 100 g^−1^)	0.24	18.20–36.10	0.45–1.03	Mahmood et al. (2018)
Moisture (g 100 g^−1^)	71.05	34.25–34.6	0.3–0.56	Fang and Ng (2015)
Ash (g 100 g^−1^)	1.19	11.05–14.2	78.1–83.7	Wall (2006)
Fiber (g 100 g^−1^)	0.65	0.61–6.5	0.29–2.7	Manaf et al. (2013)
Protein (g 100 g^−1^)	1.95	2.21–2.91	2.5–3.0	Mahmood et al. (2018)
Vitamins				
Riboflavin (mg 100 g^−1^)	0.05	3.42	21.1–25	Sirisompong et al. (2011)
Niacin (mg 100 g^−1^)	0.30	0.07	0.025	Muhtadi et al. (2016)
Thiamine (mg 100 g^−1^)	0.05	0.10	0.05	Morshed et al. (2014)

### 2.1 Nutritional composition of rambutan peels

During the processing of rambutan fruit, the peels can be seen as a large amount of waste. The peel of the rambutan fruit provides 42.1–58.7% wt, dependent on cultivar and ripeness level (Mahmood et al., 2018). The peels have higher levels of antioxidants, which are largely polyphenolic compounds and due to this reason, rambutan is widely known for its biological properties. It contains phytochemicals, e.g., corilagin, ellagic acid, and geraniin, present in the peel of this fruit, among all these, corilagin, is the most important is phytochemical (Thitilertdecha et al., 2008). In comparison with fresh peels, dried peels contained three to four times the quantity of protein and fat. This increase could damage concentration caused by humidity loss. Ascorbic acid, thiamine, riboflavin, and niacin were also present in this fruit in various quantities. Despite this, lesser levels of vitamins were found in the dry peel (Thitilertdecha et al., 2010). This might be related to vitamin heat labile when dehydrated at 55°C for 24 h. Numerous antinutritional chemicals (saponins, alkaloids, tannins, phytates, and oxalates) have been found in the peel, with corresponding concentrations of 0.51, 2.20, 1.29, 0.19, and 0.15 g 100 g^−1^. In dry peel, on the other hand, a larger concentration of each chemical. Rambutan peel, like citrus peel, pectin is present in cell wall formation. Because of its thickening property, pectin is utilized in stretches and confectionery. The fresh peel of rambutan contains 1.05–1.9 wt. % pectin, but it is black and has a low methoxy level (10.9%–11.5%). Furthermore, the pectin in the peel of rambutan has lesser solubility than pectin from pomelo. Unfortunately, due to a lack of information about cultivars and growth conditions, data comparison is impossible (Fang & Ng, 2015).

### 2.2 Nutritional composition of rambutan pulp

The pulp contains 36.7–48.5% wt. of the entire fruit and it depends on the cultivar type. The aroma of the rambutan pulp is a little bitter to sweet, along with an acidity of 2.26% and pH rates of 3.59. Six rambutan cultivars, such as Rongrien, R134, R9, Jitlee, R162, and Silengkeng, were studied to regulate their mineral content (Wall, 2006). Another study stated that the rambutan fruit can provide about 19% copper of the daily recommended intake (DRI) and 10%–11% for manganese. As with other several fruits, there is a fair quantity of vitamin C present, extending from 21.5 mg to 49.5 mg per 100 g^−1^. However, higher ascorbic acid levels (67.9–69.1 mg per 100 g) are found in Mexican vascular plant cultivars (RT‐01, RT‐02, RT‐03, RT‐04, and RT‐05). Additional vitamins, for example, thiamine, riboflavin, and niacin were also discovered. Table 1 illustrates the nutritional composition of the fruit pulp. Furthermore, some antinutritional compounds, for instance, saponins (1.49 mg per 100 g^−1^), tannin (0.13 mg per 100 g^−1^), phytates (0.14 mg per 100 g^−1^), and oxalates (0.12 mg per 100 g^−1^), have been examined (Mahmood et al., 2018).

### 2.3 Nutritional composition of rambutan seeds

The seed of rambutan is commonly considered a waste product in the processing of the fruit business, though baked seeds are safe to eat and are popular in various Asian countries. Based on cultivar and the levels of ripeness, the crushed seed have 3.9–10.1% wt. to the entire weight of a rambutan fruit (Sirisompong et al., 2011). Table 1 shows the chemical composition of fresh rambutan seed. The dried seed has increased protein, fat, and carbohydrate levels. The outline of amino acids indicates that the rambutan seeds have valuable protein contents due to the existence of many essential amino acids such as isoleucine, leucine, lysine, methionine, valine, phenylalanine, histidine and nonessential amino acids such as proline, serine, threonine, tyrosine, alanine, arginine, aspartic acid, cysteine, phenylalanine, glutamic acid, and glycine are present. In literature, there is significant diversity in lipids of seed and is dependent on cultivar, extraction circumstances, and agronomical extent of agriculture (Manaf et al., 2013). The occurrence of a significant number of lipids (18%–39%) indicates a narrative source. Arachidic acid, oleic acid, stearic acid, and palmitic acid are the primary fatty acids found in seed lipids. Again, the information of moderately substituting cocoa butter in chocolate with rambutan seed oil has become a reality.

## 3 INDUSTRIAL APPLICATIONS OF RAMBUTAN

### 3.1 Used as a food products

Rambutan fruit is frequently consumed whole when the rind is simply torn off or split in half and pulled off. On occasion, the peeled fruits are cooked and served as a dessert. They are only seldom bottled in syrup (Coronel, 1983). The fruits are often sold fresh, have a short shelf life, and are frequently canned or used to make jams and jellies. Rambutan trees are evergreen and form appealing landscape specimens thanks to their profusion of colorful fruit (Manaf et al., 2013). In Malaya, the fruit is first boiled to separate the flesh from seeds before being turned into a preserve. The testa is removed after cooling, and the seeds are cooked until tender on their own. They are blended for about 20 min with the flesh and a ton of sugar, and three cloves may be added before jars are sealed (Morton, 1987). However, these can be eaten in the Philippines just as fresh or in excess after being peeled and refrigerated to extend shelf life. Although the seeds are reportedly toxic when raw, they are occasionally roasted and consumed (Tindail, 1994).

### 3.2 Seed fat used as a cocoa replacer

In the process of making chocolate, cocoa butter is a crucial ingredient. The primary ingredient of cocoa butter, oils, and fats are triglycerides. The only persistent fat phase in chocolate products is cocoa butter as well (Lannes et al., 2003). The cost of cocoa is currently rising steadily every day. For a variety of factors, including economic and technological, researchers have been working to develop alternative fats to substitute cocoa butter in the production of chocolate (Dewettinck & Depypere, 2011). Additionally, a prior study discovered that rambutan fats (RF) are comparable to cocoa fat, despite the fact that rambutan fat has different physical characteristics, due to this reason rambutan fats can be typically used in sweets. According to a study, the fat composition in rambutan seeds ranges from 14 to 41 g/100 g (Sirisompong et al., 2011). Additionally, research on the seed has revealed that rambutan has a comparatively high quantity of fat, ranging from 17% to 39% (Morton, 1987; Zee, 1993). In addition, the demand for human consumption continued to increase in the industry. As a result, the rambutan seed's extracted fat could be a source of organic dietary fat that is suitable for industry (Solís‐Fuentes et al., 2010). SFC (solid fat content) is a significant indicator of hardness. The chocolate industry almost typically contains the fats with the lowest SFC, which results in product with softer texture since chocolate produced with softer fat has fewer crystals than chocolate manufactured with hard fat. The quantitative tendency of chocolate stiffness seemed to have an impact on the SFC profile, which also had an influence on the purified fat system's quality evaluation (Issara et al., 2014). Temperature had an influence on the quantity of solid fat in the rambutan seed fat's solid fat profile. When temperatures are low, rambutan seed fat has a softer consistency than cocoa butter, and when temperatures are high, it becomes harder. According to Solís‐Fuentes and Durán‐de‐Bazúa (2004), the compositional difference is most likely to be responsible for this behavior. As a softer filling fat compatible with cocoa butter, rambutan fat is, therefore, useful in the production of filled chocolate (Zzaman & Yang, 2013).

### 3.3 Promote shelf life of food products

One of the main causes of quality loss in edible oil is oxidative rancidity, which leads to the development of several off‐flavor compounds that render the oil unfit for human ingestion. Because of the higher proportion of unsaturated fatty acids in plant oils' structural composition, they are especially vulnerable to this quality flaw. Oils are, therefore, supplemented with various artificial antioxidants to extend shelf life and maintain flavor. In a study, sunflower oil with peel ethanolic extract was added in place of butylated‐hydroxyanisole (BHA) and tocopherols. The extract greatly increased the oil's oxidative stability compared to the control, outperformed tocopherol, but was just as effective as BHA (Mei et al., 2014).

### 3.4 Used as a thickener

As a thickening agent, flour can be added to confectionery items. In a recent study, it was found that defatted flour had lesser viscosity, solubility, and oil absorption after being treated with diluted alkali (0.075 N NaOH). On the other hand, after alkali treatment, there was an improvement in density, water permeability, swelling index, emulsification capacity, and stability. The concentration that caused the least gelation also dropped, showing that it is a great thickening and an unconventional protein source and starch (Eiamwat et al., 2016). Prior to now, rambutan seed flour (2%), a low‐calorie thickener, was used to substitute egg yolk or vegetable oil in the preparation of “Thousand Island Dressing” (Phanthanapratet et al., 2012). The prepared dressing had a calorific value that was four times lower than the standard recipe.

### 3.5 Use of rambutan as food additive

A promising ingredient for different food and pharmaceutical applications, such as a tablet binding agent or a food additive, is based on the activity of rambutan. As a food additive, rambutan seed mucilage serves as a stabilizer, thickening agent, emulsifier, texture adjuster, and fat replacement. Utilizing ethanol and water precipitation, rambutan seed mucilage (yield of 3.3% of the total) was separated (Sekar et al., 2015). Rambutan peel unveiled high antioxidant activity and was also reported to have greater amounts of flavonoid and phenolics that have the potential to work as a functional food ingredient (Riyanto & Rohman, 2017) A study by Johnson et al. (2013) reported higher ascorbic acid contents in fresh and dried pulp with lower carotene content in it.

### 3.6 Used for the encapsulation of vitamins

It was suggested that rambutan seed oil might function as a potential carrier for other bioactive substances of this kind, including fat‐soluble vitamins. Uraiwan and Satirapipathkul (2016) created nanostructured lipid carriers for vitamin E, a fat‐soluble vitamin, using the melt emulsification technique. Tween‐20 was employed as a surfactant, oil from rambutan's seed was used as a liquid lipid, stearic acid (C18:0) was used as a solid lipid, and vitamin E was trapped. Higher surfactant concentrations led to the production of smaller, more stable nanoparticles, although an optimum surfactant concentration of 5% wt. with nanoparticles measuring 139.43 nm in size was found.

### 3.7 Used as protein concentrate

**FIGURE 1:**
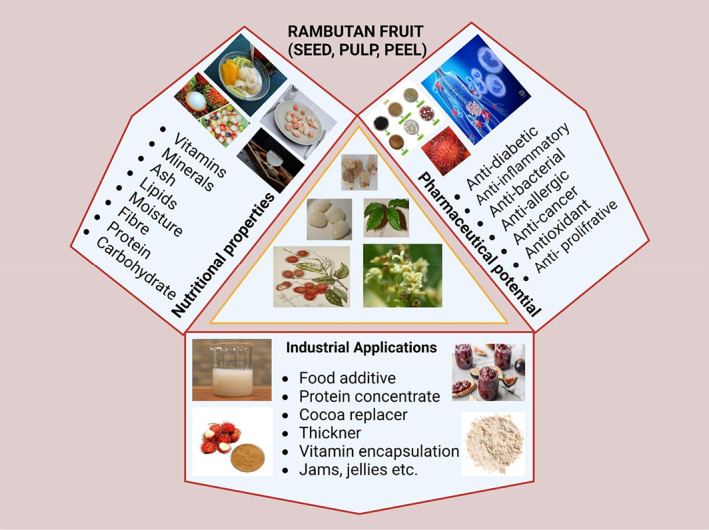
Industrial applications, nutrition properties, and pharmaceutical potential of rambutan fruit.

Protein content in rambutan seed ranges from 10.07% to 16.21%, making it a promising alternative protein source (Eiamwat et al., 2015; Fila et al., 2013). Additionally, the majority of essential and nonessential amino acids are present, which supports its highest quality (Augustin & Chua, 1988). Heat denatures the globular, water‐soluble albumin proteins. Egg white is the most typical albumin source. They carry out numerous tasks in human body, including regulating the equilibrium of colloidal osmotic pressure in the blood. Vuong et al. (2016) made an attempt to create seed albumin concentrate (80.8%) from rambutan seed lately, though. Its maximal solubility at pH 4 was discovered by further analysis. Figure 1 shows the industrial applications, nutrition properties, and pharmaceutical potential of rambutan fruit.

## 4 THERAPEUTIC POTENTIAL OF RAMBUTAN FRUIT

### 4.1 Therapeutic potential of rambutan seed

#### 4.1.1 Antioxidant activity

Rambutan seed has also been tested for antioxidant properties. It has been observed that the phenolic component concentration in rambutan seed extract is 39.55 mg of GA per 100 g. DPPH (diphenyl‐1‐picrylhydrazyl scavenging) antioxidant activity was tested with a rate of 59.16 mol Trolox per 100 g fat. This suggests using almost 39% of rambutan seed fat as a replacement for cocoa butter (Yang, 2015). The antioxidant potential of four rambutan fruit seed types has also been observed. The DPPH and ABTS, for instance, 2,2′‐azino‐bis(3‐ethylbenzothiazoline‐6‐sulfonic acid), as well as the ethyl acetate and ethanol extract from the four kinds of rambutan seed that exhibited high antioxidant potentials (Fidrianny et al., 2015). Rambutan seed extracts were observed for antioxidant potential and were investigated using the ABTS and DPPH analyses like Trolox equivalent antioxidant, and their findings were 175.08 ± 8.29 and 379.40 ± 11.01 mg g^−1^ DW, correspondingly (Chunglok et al., 2014).

#### 4.1.2 Antibacterial activity

The antibacterial properties of the extracts from the seed can be accredited to the existence of phenolics, tannins, and saponins in the seed extracts. Litchi chinensis and rambutan seed moist extract solutions were used to test the antibacterial activity of rambutan seeds. Both aqueous extracts inhibited pathogenic bacteria to a moderate extent. It also inhibited Gram‐positive strains of bacteria (*Bacillus subtilis*, *Staphylococcus aureus*, and *Streptococcus pyogenes*), as well as Gram‐negative bacterial species (*Pseudomonas aeruginosa* and *Escherichia coli*). Litchi chinensis had the best inhibitory potential as opposed to *S. pyogenes* (Bhat & Al‐daihan, 2014). The antibacterial effect of rambutan seed methanolic extracts has been investigated (roasted, raw, and boiled). The plaque diffusion assay was used to test the antibacterial action against two pathogenic strains (Gram‐positive and Gram‐negative strains) as well as for a minimum inhibitory concentration (MIC). The values showed that *Staphylococcus epidermidis* was the delicate strain of methanolic extracts of raw and boiled rambutan seeds (MIC 39 mg mL^−1^) (Rajasekaran et al., 2013).

#### 4.1.3 Antidiabetic activity

The antidiabetic effect of rambutan seed extract was observed to be high against glucosidase inhibitory activity. At a dose of 50 g, the seed extract of rambutan and the hexane fractions have been shown to have inhibiting activity as opposed to G_6_PDH and glucosidase (Soeng, 2015). In addition, the antidiabetic effect of rambutan seeds powder was also investigated at a dosage of 25 mg mL^−1^, the results indicated that rambutan seed powder was exposed to enhance glucose acceptance (Cruz et al., 2017). Another study founded on mice induced with alloxan tetrahydrate by using rambutan seed infusions has been effective in decreasing the blood sugar level and weight of mice. This seed infusion was directed to mice with a dosage of 2.85 g kg^−1^ bw which shows glucose inhibitory activity (Rahayu et al., 2013).

#### 4.1.4 Antiallergic activity

The antiallergic activity of rambutan seed extract was assessed by measuring the suppression of hexosaminidase release by basophilic leukemic cells. The release of hexosaminidase by white blood cells has been thought to be a response to allergic agents. For the result, four solvents were used to prepare the extract, atmospheric temperature water, warm water, hydro ethanol (51% ethanol), and ethanol (94%). Hexosaminidase inhibition was measured at 100 g mL^−1^ concentrations of 67.40%, 66.95%, 60.75%, and 34.75% for atmospheric temperature water, warm water, hydro ethanol, and ethanol, correspondingly (Mahmood et al., 2018).

#### 4.1.5 Antiproliferative activity

Three fruits, tamarind (Tamarindus indica), litchi (Litchi Chinensis Sonn.), and rambutan (*Nephelium lappaceum* L.), were tested for their ability to inhibit cell proliferation. These three fruit peels and seed methanolic extracts were discovered to exert an antiproliferative influence (Chunglok et al., 2014). The antiproliferative effect was identified for CLS‐354 in human mouth cancer cells. Despite the fact that the rambutan seed extract was not toxic to human cancer cells (CLS‐354) or PBMC 3, the MTT reduction assay and annexin V‐FITC/PI staining were utilized to determine chemotherapeutic and apoptosis inductive reasoning.

#### 4.1.6 Anti‐inflammatory activity

The methanol seed extract of rambutan demonstrated analgesic and anti‐inflammatory action in one study. Their seed extract exhibited 52.10% analgesic and 58.86% anti‐inflammatory action (Morshed et al., 2014). Furthermore, the antihyperalgesic efficacy of rambutan methanolic extract in raw, roasted, and boiled seeds was investigated by using the method of Eddy hot plate. The results presented that the crude methanol seed extract had stronger antinociceptive efficacy than cooked methanol seed extracts in which methanol roasted seed extracts did not display action (Rajasekaran et al., 2013).

### 4.2 Therapeutic potential of rambutan pulp

#### 4.2.1 Anti‐inflammatory and antioxidant activity

Rambutan fruit pulp has antioxidant effect that was determined using the DPPH assay, which yielded a rather poor result. Furthermore, one study found that rambutan pulp extracts had modest antioxidant activity in the ABTS and DPPH analysis when compared with rambutan peel (Chingsuwanrote et al., 2016). The anti‐inflammatory effect of ethanolic extracts of rambutan pulp was proven to utilize two kinds of rambutan fruit, such as Rongrien and Sichompu. Because of the antioxidant action of the reactive chemicals existing in both parts, the ethanolic extracts of both kinds suppressed the release of TNF but did not affect IL‐8. Table 2 discusses the detailed therapeutic potential of rambutan pulp.

**TABLE 2:** Therapeutic potential of rambutan fruit.

Therapeutic attributes	Rambutan fruit part	Therapeutic components	Functions of rambutan	References
Antioxidant	Seed	Extract of rambutan seeds	Exhibited high antioxidant	Fidrianny et al. (2015)
	Pulp	Rambutan fruit pulp	Suppressed the release of TNF‐ but no effect on IL‐8	Chingsuwanrote et al. (2016)
	Peel	Higher phenolic content	Lower pro‐oxidant capacity and having antioxidant activity	Palanisamy et al. (2008)
Antibacterial	Seed	Rambutan seed	Gram‐positive and Gram‐negative strains inhibited	Bhat and Al‐daihan (2014)
	Peel	Rambutan peel extract	Greater antibacterial action against the strains	Sekar et al. (2014)
Antidiabetic	Seed	Rambutan seed powder	Inhibitory activity opposed to G6PDH and glucosidase, also the TG extent in 3T3‐L1 cell	Rahayu et al. (2013)
	Peel	Glycogen content	Reduced lipid peroxidation and improved superoxide dismutase and glutathione peroxidase	Ma et al. (2017)
Antiallergic	Seed	Rambutan seed	Suppression of hexosaminidase	Mahmood et al. (2018)
	Pulp	Ethanolic and aqueous extracts of rambutan pulp	The inhibition of hexosaminidase	Cruz et al. (2017)
Antiproliferative	Seed	Rambutan seed	Cytotoxicity and apoptosis induction	Hernández‐Hernández et al. (2019)
	Peel	Methanolic extract of yellow rambutan peel	Have no antiproliferative action against HeLa cells	Chunglok et al. (2014)
Anti‐inflammatory	Seed	Lipopolysaccharides, peel phenolic compounds	Reduces NO content, improves mRNA levels of inducible NO synthase	Li et al. (2018)
	Peel	Lipopolysaccharides, peel phenolic compounds	Reduces NO content, improve mRNA levels of inducible NO synthase	Li et al. (2018)
Cholesterol mediate	Pulp	Rambutan fruit pulp	Drop in cholesterol level	Wall (2006)
Anticancer	Peel	Ethanolic extracts of rambutan peel	Inhibit the development of the cancer cell cycle	Cruz et al. (2017)

#### 4.2.2 Antiallergic activity

Allergy, a kind of immunological dysfunction, has appeared as a critical health effect in the age of globalization. Allergens are chemicals that elicit allergic reactions in living cells. Histamine is secreted alongside hexosaminidase after a basophil allergic response. Allergic reactions were examined in a study using ethanolic and aqueous pulp extracts. The effect of hexosaminidase was studied in basophilic leukemic cells (RBL‐2H3) (Cheong et al., 1998). The inhibited activity of hexosaminidase by ethanolic and warm water extracts was 81.96% and 85.49%, respectively. Despite this, the IC50 for warm water extract was higher (61.5 g mL^−1^) than for ethanol (31.0 μg mL^−1^) (Cruz et al., 2017).

#### 4.2.3 Cholesterol mediating effect

Cholesterol is a form of lipid that performs numerous roles in human health. Cholesterol could be categorized as either healthy or bad. Several cardiovascular problems in humans are caused by high levels of bad cholesterol. In a recent study, the impact of rambutan fruit on cholesterol‐regulating activities in humans was assessed. The rambutan fruit (350–450 g) was incorporated in an equivalent amount of both gender's (male and female) diets for 29 days. High‐density lipoproteins (HDL‐cholesterol) and serum cholesterol were recognized previous to treatment (Wall, 2006). Surprisingly, only female studies showed a substantial drop in cholesterol levels by taking supplements. Nonetheless, this reducing impact was only observed for 3 weeks, until the rambutan dosage in the diet was discontinued. The cholesterol‐lowering impact, however, was observed to be most noticeable in the group with the highest basal cholesterol in the meals. As a result, the specific mechanism requires additional investigation to lower cholesterol levels. Rambutan had been used in traditional medicine for centuries against various remedies especially as a remedy for high blood pressure and diabetes (Sukmandari et al., 2017).

### 4.3 Therapeutic potential of rambutan peel

#### 4.3.1 Antioxidant activity

Antioxidant activity is highly valued as it performs a vital role in moderating free radicals that are hazardous to a person's health. As a result, various researches reported rambutan peel extract as a possible source of antioxidants. Methanolic extract of rambutan peel has a higher antioxidant activity than butylated hydroxytoluene (BHT). The lipid peroxidation activity (75–190 times) and DPPH (40–90 times) assay results were higher than the BHT. From this research, it can be noted that the DPPH and ABTS analysis indicated IC50 (g mL^−1^) rates of the methanolic peel extracts (Thitilertdecha et al., 2010). Another study demonstrated a higher phenolic content, lower pro‐oxidation capability, and a strong antioxidant effect in peel extract of rambutan (Palanisamy et al., 2008). Table 2 discusses the therapeutic potential of rambutan peel in detail.

#### 4.3.2 Antibacterial activity

Researchers had examined the antimicrobial potential of rambutan fruit wastes under the natural antibacterial agents (Thitilertdecha et al., 2008). Thitilertdecha conducted a relation of rambutan seeds along with their peel extracts, by analyzing eight pathogenic bacterial strains (*Escherichia coli*, *Staphylococcus aureus*, *Klebsiella pneumoniae*, *Salmonella typhi*, *Pseudomonas aeruginosa*, *Vibrio cholera*, *Staphylococcus epidermidis*, and *Enterococcus faecalis*). When rambutan peel extract and seed were examined, the peel showed greater antibacterial action against the strains than the seed. An antagonistic property was shown by *Vibrio cholerae*, *Pseudomonas aeruginosa*, *Staphylococcus epidermidis*, *Enterococcus faecalis*, and *Staphylococcus aureus* observed toward pathogenic bacterial strains, and showed an antagonistic property toward bacteria, the remaining three bacteria had not been found under antibacterial activity (Chunglok et al., 2014).

According to Malini and Maheshkumar (2013), rambutan peel extracts exhibit the highest antibacterial activity against the *P. aeruginosa* species and the lowest activity against *C. tetani*. Two varieties of the methanolic extracts of red and yellow rambutan peels were also examined, the red rambutan peels indicated less activity as compared to the yellow rambutan peel in contradiction to *Staphylococcus aureus* and *Streptococcus pyogenes* with inhibitory zones of 6–9 and 6–13 mm individually, having diverse concentrations. However, neither peel nor extract of rambutan inhibited the *E. coli* nor *P. aeruginosa* (Sekar et al., 2014).

The rambutan peel extract showed an antibacterial action toward *Streptococcus mutans* (ATCC 25175) and *Staphylococcus aureus* (ATCC 6538), but had not shown an antagonistic activity toward Gram‐negative strains (*Candida albicans*, *E. coli*, *Pseudomonas aeruginosa*, *S. typhi*, and *K. pneumoniae*).

In AgNP production, rambutan peel extract was used as an initiator at room temperature in an aqueous medium. The creation of AgNP was demonstrated by an examination of the Ultraviolet–Visible spectrum, and SEM‐energy‐dispersive X‐ray (SEM‐EDS) used to demonstrate that AgNP was produced in the same way with a wide particle size distribution 50 L of AgNP demonstrated antibacterial efficacy against *Salmonella paratyphi* (Lestari et al., 2014).

#### 4.3.3 Antidiabetic activity

**FIGURE 2:**
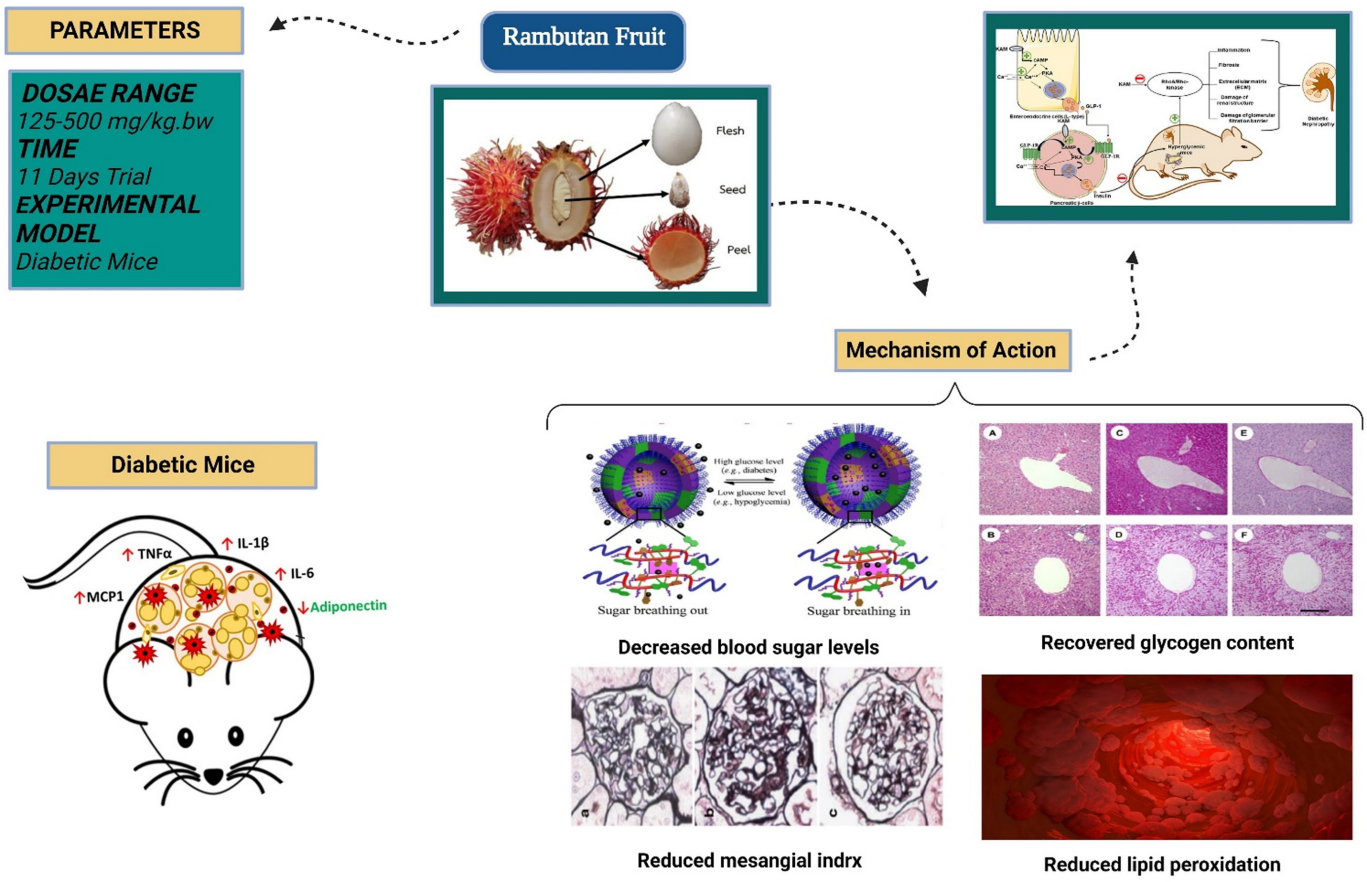
The therapeutic potential of rambutan fruit in diabetic mice.

Many studies on the applications of rambutan peels have recently been conducted and described that the rambutan peel extract is an herbal remedy for the treatment of obesity. As a result, various researches have revealed that rambutan peel extract possesses antidiabetic potential. Rambutan peel extract's ability to treat type 2 diabetes in mice was investigated and is shown in Figure 2. The study found that the peel extract of rambutan boosted body weight while it decreases blood sugar levels. It also measures the total cholesterol level, lipids, and serum protein levels in diabetic mice in a dose‐dependent manner. By using rambutan peel extract, the glycogen content was recovered in mice livers (Ma et al., 2017). Moreover, in diabetic mice, it reduced lipid peroxide levels and improves the activity of superoxide dismutase and glutathione peroxidase. Additionally, the research revealed that rambutan peel extracts efficiently protect the pancreas, liver, and kidney tissue structure and reduce the risk of mesangial index.

An 11‐day research study having durian (*Durio zibethinus* Murr.) and rambutan (*Nephelium lappaceum* L.) peel extracts in mice diagnosed with diabetes at dosages of 120, 260, and 500 mg kg^−1^ bw was conducted. Doses range from 120 to 500 mg kg^−1^ bw, and rambutan and durian peel extracts showed an antidiabetic action. In a model of diabetic mice, standardized with geraniin, the antidiabetic properties of rambutan peel extract were examined to (Muhtadi et al., 2016) establish type 2 diabetes, male mice were given a fatty diet and injected with 220 mg kg^−1^ nicotinamide and 60 mg kg^−1^ streptozotocin. For 28 days, diabetic mice were given the peel of rambutan extract at doses of 450 and 2000 mg. The positive control rats were given 210 mg of metformin. This study found that diabetic mice given 2000 mg of rambutan peel had lower blood sugar levels and better insulin levels, alike the group given metformin. The pancreas histology revealed that the group given 2000 mg of rambutan peel extract has healthy pancreatic activities than the metformin treatment group (Subramaniam et al., 2015).

#### 4.3.4 Antiproliferative activity

In one study on rambutan peel, the antiproliferative effect was determined for cervical cancer cells (HeLa), breast cancer cells (MDA‐MB‐231), and osteosarcoma cancer cells (MG‐63). Rambutan varieties such as yellow and red were used for the study. The methylene blue test and cisplatin were used to assess the cytotoxicity in vitro for the extract of the peel of rambutan to a standard MDCK cell line. MDAMB231 and MG63 were both inhibited by the extract of methanolic content of yellow peel of rambutan, with IC50 values of 4.92 ± 1.71 and 7.01 ± 1.01 g mL^−1^, correspondingly. Both rambutans have no antiproliferative properties contrary to He.La cells (Khaizil Emylia et al., 2013). The peel‐ and seed‐extracted solutions of three tropical fruits, rambutan, litchi, and tamarind, were also tested for antiproliferative activity. The MTT reduction assay and annexin V409 FITC/PI staining were used to assess cytotoxicity and apoptotic induction. The seed extract of tamarind was cytotoxic to human oral cancer (cells ‐354); however, rambutan (peel and seed extracts) and litchi were not poisonous to CLS‐354 or PBMC (Chunglok et al., 2014).

Another study conducted on the antiproliferative properties of extracts of coconut peel, rambutan, and mangosteen for Caco‐2 (human colorectal adenocarcinoma) and KB lines (epidermal carcinoma of the oral cavity with contamination HeLa) revealed that coconut peel extract was cytotoxic to the KB cell line, whereas rambutan and mangosteen had no cytotoxic impact on either cell (Khonkarn et al., 2010).

#### 4.3.5 Anti‐inflammatory activity

Because of their ability to eliminate free radicals, phenolic chemicals found in plants have been researched. It showed the protection of cells from degeneration and has a significant potential for anti‐inflammation effect. The anti‐inflammatory effect was investigated using RAW cells (264.7) generated by rambutan peel phenolic compounds (RPP) and lipopolysaccharides (LPS) over the appearance of the inducible nitric oxide (iNOS) gene, and secretions of nitric oxide concentrations were calculated, the final result demonstrated that the production of RPP at 395 g mL^−1^ of iNOS substantially reduced the contents of NO, decreasing by 40%, while also enhanced the levels of mRNA to iNOS synthase in RAW 264.7 cells caused by LPS (Li et al., 2018). Rambutans have an anti‐inflammatory effect and it was observed by using ethanolic extract solutions of all portions of the fruit and observed that both species of rambutan suppressed TNF‐ but has no effect on IL‐8 secretion (Chingsuwanrote et al., 2016).

#### 4.3.6 Anticancer activity

Recently, researchers have found evidence that reactive oxygen species (ROS) had a role in the evolution of cancerous cells, on the other hand, the bioactive chemicals demonstrate a significant interest. The rambutan peel has been proven to exhibit antibacterial properties to prevent cancer cell growth. Thirteen Malaysian plants, including rambutan, were also evaluated. At dosages of 50 and 100 g mL^−1^, the aqueous and methanolic rambutan peel extracts had no antileukemic effects on 4T1 and 3T3 cells (Ling et al., 2012). In vitro activity against human osteogenic sarcoma and cancerous cells were conducted which indicated that ethanolic peel extracts showed positive results. Yet, it did not affect normal cells. However, the rambutan peel extract prompted G2/M seizure by hindering the development of the cancerous cell cycle (Cruz et al., 2017).

## 5 CONCLUSION

The investigations on the rambutan fruit, pulp, and seeds have confirmed that it contains a number of bioactive components and is an essential commercial fruit. Numerous studies have suggested the presence of bioactive components in the seeds, peels, and pulp that have nutritional properties and therapeutic potential such as antioxidant, antibacterial, anticancer, antidiabetic, antiviral, anti‐inflammatory, and antiproliferative activities. The review explains the potential of edible and nonedible portion of rambutan fruit in various value‐added functional food. However, by using proper protocols and processing technologies, rambutan fruit along with its by‐products can be utilized as a functional component in numerous low‐cost food products. However, more research can be done on the underutilization of rambutan fruit to seek more possibilities.

## AUTHOR CONTRIBUTIONS

**Muhammad Afzaal:** Conceptualization (equal); investigation (equal). **Farhan Saeed:** Conceptualization (equal); data curation (equal). **Maryam Bibi:** Validation (equal). **Afaf Ejaz:** Investigation (equal). **Yasir Abbas Shah:** Visualization (equal); writing – original draft (equal). **Zargham Faisal:** Validation (equal). **Huda Ateeq:** Software (equal); writing – review and editing (equal). **Noor Akram:** Writing – review and editing (equal). **Aasma Asghar:** Software (equal). **Mohd Asif Shah:** Visualization (equal); writing – review and editing (equal).

## CONFLICT OF INTEREST STATEMENT

The authors declare no conflict of interest.

## ETHICS STATEMENT

This article does not contain any human participants and animal‐based studies.

## Data Availability

Data will be provided on request.
